# The Added Burden of Personality Disorder on Subsidized Australian Health Service Utilization Among Women With Mental State Disorder

**DOI:** 10.3389/fgwh.2021.615057

**Published:** 2021-03-23

**Authors:** Bianca E. Kavanagh, Stella M. Gwini, Julie A. Pasco, Amanda L. Stuart, Shae E. Quirk, James Gaston, Kara L. Holloway-Kew, Alyna Turner, Michael Berk, Olivia M. Dean, Andrew M. Chanen, Heli Koivumaa-Honkanen, Paul Moran, Rohan Borschmann, Lana J. Williams

**Affiliations:** ^1^School of Medicine, Institute for Physical and Mental Health and Clinical Translation, Barwon Health, Deakin University, Geelong, VIC, Australia; ^2^University Hospital Geelong, Barwon Health, Geelong, VIC, Australia; ^3^Department of Epidemiology and Preventive Medicine, Monash University, Melbourne, VIC, Australia; ^4^Department of Medicine-Western Health, University of Melbourne, St. Albans, VIC, Australia; ^5^Institute of Clinical Medicine/Psychiatry, University of Eastern Finland, Kuopio, Finland; ^6^Institute of Clinical Medicine, Kuopio Musculoskeletal Research Unit (KMRU), University of Eastern Finland, Kuopio, Finland; ^7^Faculty of Health and Medicine, School of Medicine and Public Health, The University of Newcastle, Callaghan, NSW, Australia; ^8^Florey Institute for Neuroscience and Mental Health, University of Melbourne, Parkville, VIC, Australia; ^9^Department of Psychiatry, University of Melbourne, Parkville, VIC, Australia; ^10^Orygen, Parkville, VIC, Australia; ^11^Centre for Youth Mental Health, The University of Melbourne, Parkville, VIC, Australia; ^12^Department of Psychiatry, Kuopio University Hospital, Kuopio, Finland; ^13^Population Health Sciences Department, Centre for Academic Mental Health, Bristol Medical School, University of Bristol, Bristol, United Kingdom; ^14^Justice Health Unit, Centre for Health Equity, Melbourne School of Population and Global Health, The University of Melbourne, Melbourne, VIC, Australia; ^15^Centre for Adolescent Health, Murdoch Children's Research Institute, Melbourne, VIC, Australia; ^16^Health Service and Population Research Department, Institute of Psychiatry, Psychology, and Neuroscience, King's College London, London, United Kingdom; ^17^Melbourne School of Psychological Sciences, The University of Melbourne, Melbourne, VIC, Australia

**Keywords:** personality disorder (MeSH), mental state disorder, health service utilization, mental disorder, psychiatry

## Abstract

This study aimed to investigate health service utilization among women with mental state disorder only (MSD-PD), mental state disorder plus personality disorder (MSD+PD), and controls in a population-based sample. Women (*n* = 635) from the Geelong Osteoporosis Study completed mental health assessments and were categorized into groups (MSD-PD, MSD+PD, controls). General practitioner (mental and non-mental health encounters) and specialized mental health service utilization was ascertained from data linkage to the Medicare Benefits Schedule, Australia (01/09/2008-31/12/2012). Negative binomial and binary logistic regression models were employed to assess health service utilization differences between groups. Results indicated that women with MSD+PD had more encounters of non-mental health service utilization than women with MSD-PD and controls. Age significantly modified these relationships: women with MSD+PD and MSD-PD had more encounters of health service utilization at midlife and in the seventh decade of life. No significant differences were found in the frequency of general practitioner mental health service utilization or specialized mental health service utilization between groups. These data suggest that the presence of co-occurring PD is associated with increased health service utilization among women with other common mental health problems. Healthcare providers should be vigilant to the presence of PD when establishing management plans with patients presenting with common mental health problems.

## Introduction

Personality disorder (PD) is a common disorder, affecting about one-in-eight individuals in the community globally (1). By definition, PD occurs when an individual's personality structure inhibits them from attaining adaptive solutions to general life tasks, and this is relatively stable across time and situations (2). PD commonly co-occurs with mental state disorder (MSD), which refers to disorders of episodic disruptions of mental state, such as mood, anxiety, eating, and substance use disorders. Furthermore, individuals with PD and MSD commonly experience co-occurring physical conditions, and this co-occurrence can significantly increase functional disability, poor health outcomes, and health service utilization (HSU) (3).

Both MSDs and PDs have been independently associated with increased HSU, especially emergency department visits and inpatient hospitalizations (4–6). Some research has shown that MSDs mediate the relationship between PD and increased utilization of general practitioner (GP) services (7). Similarly, Coid et al. (8) found that, of the types of HSU contacts studied (i.e., GP, psychiatrist, psychologist, social worker, community/other nurse, and self-help group), contacts with GPs were the only kind of health consultation sought by individuals with borderline PD, independent of co-occurring MSDs and other socio-demographic correlates. However, the nature of these consultations (i.e., physical or mental health-related encounters) was not reported in that study. Still, higher usage of psychosocial and outpatient psychotherapy services, psychotropic medications, and psychiatric hospitalizations has been documented in individuals with PD compared to those with depression (9). Therefore, it appears that the nature and size of association between MSDs, PD, and HSU remains insufficiently understood.

There are robust data to suggest that women utilize healthcare services, particularly GP consultations and mental health related services more than men (10). These patterns remain even after controlling for sex-specific factors, such as post-menopausal hormone therapy and gravidity (11). However, the potential combined effects that MSDs and PD may have on HSU among women has not been well-studied, despite the high rate of co-occurrence [~50%; (12)] amongst this group in the clinical setting. This has important ramifications for clinical priorities and healthcare management. Thus, further examination of HSU of women in non-treatment seeking samples is needed.

With these issues in mind, we set out to examine differential patterns of HSU associated with MSD and PD in an ongoing cohort of adult women. We hypothesized that women with MSD+PD would have more HSU encounters than women with MSD-PD and women with no history of these disorders.

## Methods

This research was undertaken in accordance with the latest version of the Declaration of Helsinki and approved by the Barwon Health Research Ethics Committee (Reference No. 92/01_E7). Written informed consent was obtained by all participants in this study.

### Participants

Participants were women from the Geelong Osteoporosis Study (GOS). The GOS is an age-stratified, population-based study of women randomly selected from the electoral roll for the Barwon Statistical Division, located in south-eastern Australia. Details of GOS have been published previously (13). Briefly, 1494 women (aged between 20 and 94 years) initially participated in the study and have since been invited to take part in follow-up assessments every 2–5 years. In 2005 an additional 246 women (aged between 20 and 30 years) were recruited into the study, with at least 100 women in each 5-year age stratum to afford the full age range to be explored. All data pertaining to the current study were collected during the 2011–2014 follow up phase of the GOS. Participants who did not complete a psychiatric assessment (*n* = 81) or did not have Medicare Benefits Schedule (MBS) data available (*n* = 99) were excluded from the analyses. Women without co-occurring MSD (*n* = 34) were also excluded as the primary aim was to examine the impact of presence/absence of PD on women with MSD, resulting in a final sample of *n* = 635.

### Measures

#### Outcome

Health service utilization data were obtained via linkage with the Medicare Benefits Schedule (MBS), Australia. The MBS is a list of subsidized medical and hospital services, provided through the Australian public health insurance system, known as Medicare (14). The MBS includes services provided by GPs, recognized specialists, consultant physicians; tests and examinations conducted by doctors to diagnose and treat illnesses; most surgical and therapeutic procedures performed by doctors; some approved dental services; optometry examinations; and particular services provided by allied health professionals (14). The MBS however, does not capture data on public hospital inpatient admissions (except for those accessing private health insurance), mental health inpatient admissions, community mental services provided by the state, or dental services performed on most adults (with the exception of individuals eligible through Centrelink benefits).

Data linkage to the MBS was performed for each GOS participant over a 4.33-year period (01/09/2008-31/12/2012), which is the maximum length of time MBS data may be requested per individual. Data linkage was facilitated and overseen by Services Australia (formerly the Department of Human Services) using pertinent information collected from the relevant consent forms and a unique ID to enable linkage. We were interested in data pertaining to non-mental health services, including GP, dental, optician or optometrist services, and allied health services (allied health services include encounters of diabetes education, exercise physiology and dietetics services); and mental health services subsidized by Medicare, including GP mental health treatment, and services provided by psychiatrists, clinical psychologists, and general psychologists. Health services were grouped as follows: (i) non-mental health services (i.e., GP non-mental health service consultations, dental services, optometrist or optometry services, and allied health), (ii) GP mental health services, and (iii) specialized mental health services (i.e., psychiatry and psychology services). For the purposes of this study, HSU was defined as an MBS recorded encounter within the healthcare system.

#### Exposures

Lifetime history of MSD (mood, anxiety, substance use, and eating disorders) was determined using the Structured Clinical Interview for DSM (SCID) Axis I Disorders, non-patient edition (SCID-I/NP) (15). The SCID-I captures MSD diagnoses that are current at the time of interview, as well as past diagnoses. For the purposes of the present study, lifetime history was determined as a current or past MSD diagnosis. PD was assessed using the SCID Axis II Disorders (SCID-II) (16). The SCID-I and SCID-II have demonstrated adequate reliability ranges for categorical diagnoses using joint interviewer and observer methods (17, 18). Reliability coefficients for the SCID-I have been shown to be moderate for major depression (κ = 0.61), substance use (κ = 0.76–0.77), and eating disorders (κ = 0.64), and weak to moderate for anxiety disorders (κ = 0.44–0.78) (18). The SCID-II has yielded strong reliability coefficients for dependent (κ = 0.86) and obsessive-compulsive (κ = 0.83) PDs and excellent reliability coefficients for paranoid (κ = 0.93), schizoid (κ = 0.91), schizotypal (κ = 0.91), antisocial (κ = 0.95), borderline (κ = 0.91), histrionic (κ = 0.91), narcissistic (κ = 0.98), and avoidant (κ = 0.97) PDs (17). Trained researchers with psychology qualifications who had undertaken comprehensive training concordant with the SCID protocols completed interviews. Participants were classified into one of the following categories: (i) lifetime MSD-PD; (ii) lifetime MSD+PD; and (iii) no history of any MSD or PD (denoted as controls hereafter).

Area-based socio-economic status (SES) was identified using the Index of Relative Socioeconomic Advantage and Disadvantage (IRSAD) (19). Greater SES disadvantage is depicted by lower quintiles of the IRSAD (Quintile 1). Habitual physical activity was determined by classifying participants' responses (active/inactive) to survey items regarding light to vigorous physical activity. Weight (kg) and height (cm) were measured and used to determine body mass index (BMI). Participants were asked to self-report the presence of physical conditions, and medical records, medication use, or clinical data confirmed these where possible. A modified Charlson Comorbidity Index (CCI), based on chronic physical diseases (20), was calculated for each participant.

### Statistical Analysis

All statistical analyses were planned *a priori* and performed using STATA IC version 16 (21). Categorical data were reported using frequencies and percentages. Kruskal-Wallis and Chi-square-tests were used to examine differences in demographic characteristics between groups (MSD-PD, MSD+PD, controls). These data were reported using medians with the interquartile range (IQR, 25th and 75th percentiles). Statistical significance was set at α = 0.05.

Negative binomial regression [incident risk ratios (IRR) with 95% confidence intervals (CI)] was employed to account for the over-dispersed count HSU data. This modeling was used to examine the association between the three groups and non-mental HSU, with the control group set as the reference. Differences between MSD-PD and MSD+PD were then investigated, with MSD-PD set as the reference. Covariates including age, SES, BMI, physical activity and CCI score were tested for inclusion in the models. Interaction terms were checked for effect modification. A subgroup analysis utilizing binary logistic regression [odds ratios (ORs) with 95% CI] was then conducted to determine whether the two groups, MSD-PD and MSD+PD differed with regards to the following dichotomized outcomes (i) GP mental HSU (yes/no) and (ii) specialized mental HSU (yes/no).

## Results

Two hundred and eleven (33.2%) women met criteria for having a lifetime history of MSD-PD, 103 (16.2%) women were identified as having MSD+PD, and 321 (50.6%) women had no history of MSD or PD. Of the women who met criteria for PD within the MSD+PD group, 30 (29.1%) women had a Cluster A PD, 16 (15.5%) women had a Cluster B PD, and 79 (76.7%) women had a Cluster C PD. There was also comorbidity across the clusters, with four (3.9%) women meeting criteria for a PD from each of the three clusters and 16 (15.5%) women meeting criteria across two clusters. Mood disorders were the most frequently diagnosed lifetime MSD (*n* = 255, 81.2%). They were followed by anxiety (*n* = 164, 52.2%), eating (*n* = 32, 10.2%) alcohol use (*n* = 15, 4.8%), and substance use disorders (*n* = 15, 4.8%). Participants may have met diagnostic criteria for more than one type of PD or MSD, and therefore, these characteristics data are not mutually exclusive. There were differences between the groups in age; otherwise they were similar in SES, physical activity, and BMI. Characteristics of the whole group and for women with MSD-PD, MSD+PD and controls are displayed in Table 1.

**Table 1:** Characteristics of the study group and women with MSD-PD, MSD+PD, and controls.

**Characteristic**	**All (*n =* 635)**	**MSD-PD (*n =* 211)**	**MSD+PD (*n =* 103)**	**Controls (*n =* 321)**	***p***
Age (years)	56.5 (42.4–67.5)	55.2 (39.1–64.5)	52.9 (38.3–63.2)	60.0 (45.7–71.5)	**0.001**
SES					0.141
Quintile 1^a^	98 (15.4%)	36 (17.1%)	19 (18.5%)	43 (13.4%)	0.155
Quintile 2	73 (11.5%)	30 (14.2%)	12 (11.7%)	31 (9.7%)	
Quintile 3	245 (38.6%)	77 (36.5%)	34 (33.0%)	134 (41.7%)	
Quintile 4	113 (17.8%)	42 (19.9%)	21 (20.4%)	50 (15.6%)	
Quintile 5	106 (16.7%)	26 (12.3%)	17 (16.5%)	63 (19.6%)	
BMI (kg/m^2^)^b^	27.1 (23.7–32.0)	26.9 (23.5–31.2)	28.4 (24.2–33.7)	27.0 (24.0–31.9)	0.203
Physical activity (active)^c^	162 (25.8%)	48 (22.8%)	31 (30.4%)	83 (26.4%)	0.333
HSU					
Non-mental health (all encounters)	20 (14–28)	20 (15–29)	21 (15–30)	19 (13–26)	**0.009**
Non-mental health (≥1 encounter)	626 (98.6%)	208 (98.6%)	102 (99.0%)	316 (98.4%)	0.908
GP mental health (≥1 encounter)	107 (16.9%)	59 (28.0%)	30 (29.1%)	18 (5.6%)	**<0.001**
Specialized mental health (≥1 encounter)	70 (11.0%)	37 (17.5%)	24 (23.3%)	9 (2.8%)	**<0.001**

*Values reported as frequency (%) or median (interquartile range)*.

a *Greatest disadvantage*.

b *BMI n=617*.

c *Physical activity n = 628*.

*MSD, Mental state disorder; PD, Personality Disorder; SES, Socio Economic Status; BMI, Body Mass Index. Bold values indicate the significant results*.

**Figure 1:**
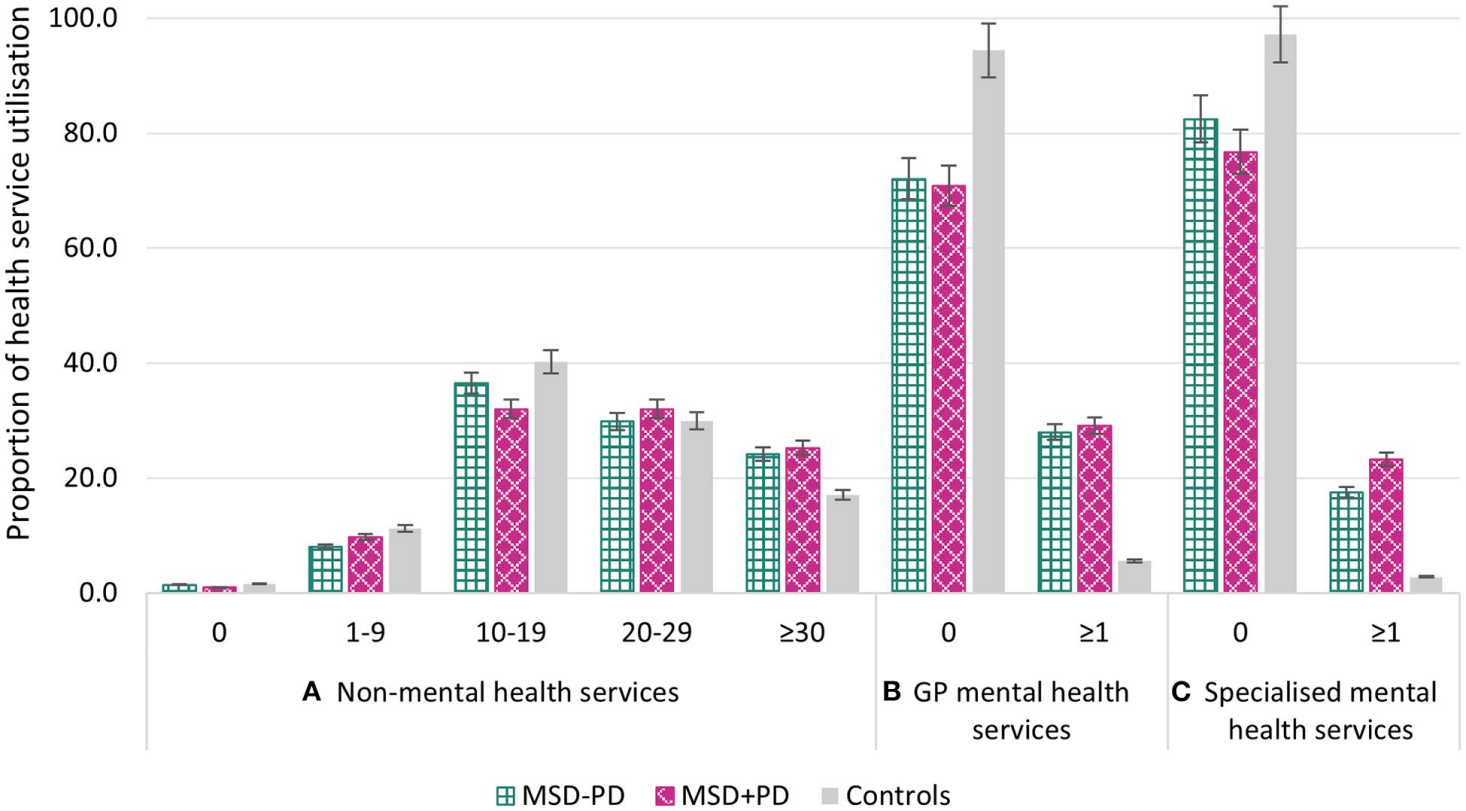
**(A–C)** Proportion of health service utilization by its type and across the sample. MSD, Mental state disorder; PD, Personality disorder.

Over the 52-month study period, the average number of HSU encounters per participant was 23.46 (*SD* = 17.68) for non-mental HSU, 0.37 (*SD* = 1.12) for GP mental HSU, and 1.48 (*SD* = 7.71) for specialized mental HSU. Figure 1 presents proportions of HSU between the study groups.

Encounters for non-mental HSU differed between the women with MSD-PD, MSD+PD, and controls [25.4 (*SD* = 19.8) vs. 26.5 (*SD* = 21.9) vs. 21.1 (*SD* = 14.1), *P* = 0.009]. After adjustment for age, SES, BMI, physical activity and CCI score, non-mental HSU was greater among women with MSD-PD [adjusted IRR 1.85 (1.25-2.74-2.61), *P* = 0.002] and women with MSD+PD [adjusted IRR 3.48 (2.16–5.61), *p* < 0.001] when comparing both to controls. Similarly, encounters of non-mental HSU were greater for women with MSD+PD compared to women with MSD-PD [adjusted IRR 1.88 (1.13–3.11), *P* = 0.015].

**Figure 2:**
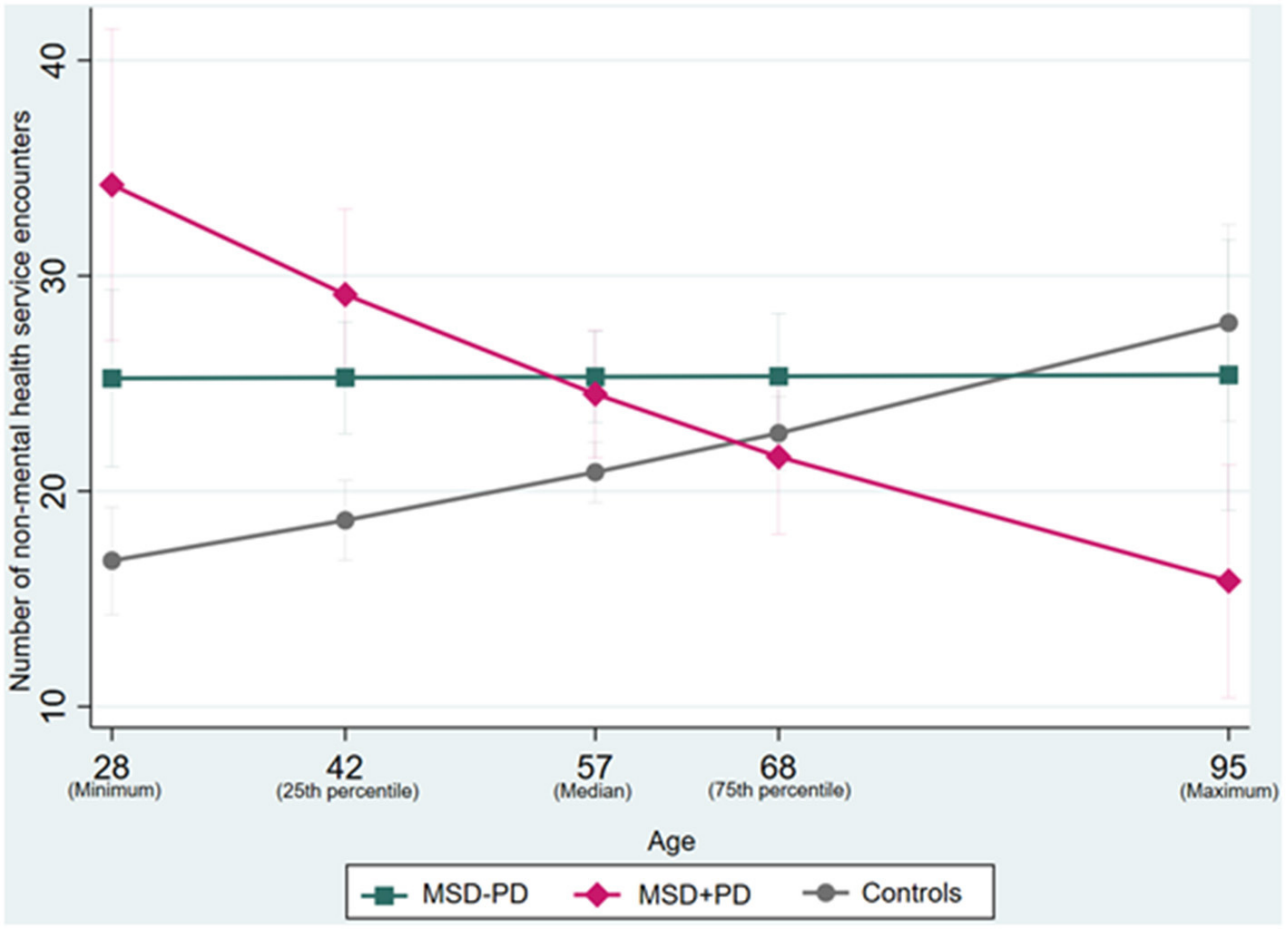
Interaction effects between age and study groups status for non-mental health service utilization. MSD, Mental state disorder; PD, Personality Disorder.

Age was found to be an effect modifier in the association between mental disorder status and non-mental HSU (Figure 2). Encounters of non-mental HSU differed between the groups at the 25th percentile of median age (42 years) for women with MSD-PD [estimated mean difference = 6.63, (95% CI 3.52–9.73), *P* < 0.001] and MSD+PD [estimated mean difference = 10.48, (95% CI 6.08–14.88), *P* < 0.001], compared with controls. A difference was also observed at median age (57 years) for women with MSD-PD [estimated mean difference = 4.43, (95% CI 1.89–6.97), *P* = 0.001] and MSD+PD [estimated mean difference = 3.63, (95% CI 0.33–6.93), *P* = 0.31], compared with controls. No differences in encounters of non-mental HSU was apparent at the 75th percentile of median age (68 years) for women with MSD-PD [estimated mean difference = 2.65, (95% CI −0.76–6.05), *P* = 0.127] and MSD+PD [estimated mean difference = −1.09, (95% CI −5.06–2.88), *P* = 0.590], compared with controls.

In a subgroup analysis, encounters of GP mental HSU did not differ between women with MSD-PD and MSD+PD (28.0 vs. 29.1%, *P* = 0.830). Further adjustment for age did not affect the relationship [adjusted OR 1.03 (0.60–1.76), *P* = 0.920]. Likewise, encounters of specialized mental HSU did not differ between women with MSD-PD and MSD+PD (17.5 vs. 23.3%, *P* = 0.227), with adjustment for age not affecting the relationship [adjusted OR 1.42 (0.77–2.61), *P* = 0.257].

## Discussion

This study aimed to examine utilization of subsidized MBS health services among women with MSD-PD, MSD+PD, and with no history of these disorders. Results indicated, partially concordant with the hypothesis, that women with MSD+PD had more encounters of non-mental HSU compared to women with MSD-PD and controls. Importantly, however, the results of this study must be considered in relation to the limitation that MBS data do not capture the full extent of HSU, especially with regard to encounters of mental HSU, for example encounters of mental health inpatient admissions and community mental health services provided by the state. Nonetheless, it appears that those with mental disorders had encounters with non-mental health professionals for reasons that were not directly recorded as being mental health-related, nor explained by other relevant health factors (i.e., SES, BMI, physical activity, and mortality risk associated with chronic physical conditions).

The results of this study are partly in keeping with the wider literature reporting that individuals with PD utilize some health services, such as GP services, more than those with MSDs and the general population (7–9). In Australia, GPs (included in our non-mental health service group) are generally the first encounter an individual has with the health system and this service is the gateway to gaining access to specialist care (10). Thus, those with mental disorders, particularly those with PD, may seek recurrent encounters with GPs or other non-mental health care providers to obtain access to secondary or tertiary health services, a notion that those with no history of these disorders do not appear to replicate to the same extent.

It is well-known that individuals diagnosed with PD experience a range of complex and often enduring psychosocial difficulties (22), and so women with MSD+PD may have frequent encounters with GPs (for non-direct mental health reasons), reflecting these issues. Moreover, individuals diagnosed with cluster C PD (i.e., individuals with these disorders have traditionally been denoted as being anxious or fearful) formed the largest group in the current sample and it should be emphasized that this group are predominately treatment-seeking (as opposed to treatment-rejecting) (23). Particular factors which are pertinent to seeking treatment for PD diagnosis, such as motivation to change and willingness to undertake psychopharmacological treatments (23), may also be relevant to seeking treatment in non-mental health settings.

The current study also showed that differences in encounters of non-mental HSU were greatest amongst younger age groups, but interestingly this difference was not apparent among older women (>68 years). Although caveated by the types of health services assessed in the current study, these data reflect the concerning notion that older adults are considerably less likely to seek mental health help than middle-aged adults (24), which may also be pertinent to non-mental HSU. This may be especially true for those with PD, of which previous research has shown that individuals with PD tend to be younger and experience more disability than individuals without PD (25). However, little research has specifically examined health outcomes and their consequences on HSU of those with PD in later life, though a recent review has emphasized the need for research in this area (26).

For GP mental HSU and specialized mental HSU, no differences among women with MSD-PD and MSD+PD were found. Recent Australian data has shown that GPs were more commonly encountered for reasons pertaining to depression, compared to encounters for type II diabetes and other mental health conditions (27), though it should be noted that PD was not included in this previous study. Mood disorders were the most frequently diagnosed disorder among those with an MSD (81.2% prevalence) and thus may be a key driving factor for GP mental health encounters for both women with MSD-PD and MSD+PD. Furthermore, women with anxiety disorders and Cluster C PDs were common in this sample. These mental disorders have been linked with anxious/insecure attachment styles (28, 29), which may in themselves lead to high utilization of GP and mental health services (30). This may have diluted the ability to detect true differences in GP and specialized mental health service utilization between the MSD-PD and MSD+PD groups. Moreover, this finding is caveated by the confines of the current dataset and is in contrast with previous research, which has predominately used samples with younger adults (9, 31).

### Strengths and Limitations

This study has several strengths, including being comprised of a large, population-based random sample of women, using a prospective cohort design. Data on mental disorders were assessed using the SCID, which is a widely accepted, rigorous, standardized assessment. This study included government-captured HSU data, which is not readily available in many countries. This method provides an accurate representation of real-life encounters of HSU. It is noteworthy that nearly all of our sample (98.6%) had a non-mental health encounter (i.e., GP, dental, optician or optometrist services, and allied health services) at least once during this study period (52 months), and this is consistent with data pertaining to high rates (96.1%) of GP consultations within the Australian population (10). These strengths extend the literature pertaining to women's mental health and the use of the Australian healthcare system by women.

Despite the strengths, the current study has some important limitations. Data on hospitalizations, visits to emergency departments, private healthcare use, and utilization of non-conventional mental health services (e.g., online or telephone helplines) are not included in MBS data. Moreover, data on MBS mental HSU encounters do not include individuals who choose not to claim for subsidized payments or those accessing services provided by the state psychiatric system, which captures individuals with some of the most severe mental disorders. This limitation may be reflected in the low counts of HSU across the sample, which precluded analyses on specific HSU categories from being performed. This also resulted in some relevant services (e.g., services provided by social workers and occupational therapists) having to be excluded from analyses. Health service utilization data were collected via the Australian healthcare system and current findings may not be generalizable to other countries due to potential cultural and healthcare system differences. Additionally, the data in the current study were cross-sectional, preventing the examination of HSU over time. Further, the youngest participant in the current study was 28 years old, and our findings may only apply to middle-aged and older women. However, this is an area of research which requires urgent attention, with a recent review highlighting the need for clinicians to be aware of PD in older adults (26). There may also be differences in psychiatric profiles between responders and non-responders in the current study. We are unable to eliminate the possibility of differential loss to follow-up throughout the study, particularly concerning PD status. Lastly, there were very few participants who met criteria for Cluster B PDs and encounters of HSU may have been understated due to this. Future research should extend this line of enquiry and consider investigating the added burden of specific types/clusters of PD on HSU.

### Conclusion

In conclusion and with respect to the limitations of the data, this study shows that women with MSD+PD have more frequent encounters of non-mental HSU than women with MSD-PD or controls without a history of any mental disorder. The focus of providing appropriate and timely treatments should be a paramount consideration for professionals who do not specialize in mental health settings. Given difficulties in accessing appropriate care and treatment, individuals with PD may be motivated/or pushed to seek treatment in non-mental health settings. Healthcare professionals should be aware of the implications of this on treatment management broadly, especially considering the high mortality rate for those with PD, evidenced in other research (32). This study did not find evidence for higher utilization of specialized mental health services between those with MSD-PD and MSD+PD, though this may be explained by the lack of hospital inpatient admission data. Nonetheless, these data highlight that the presence of co-occurring PD is associated with increased HSU in some circumstances among women with other common mental health problems. These findings may be helpful for healthcare professionals to be aware of PD when managing patients who present with common mental health problems.

## Data Availability Statement

The data analyzed in this study is subject to the following licenses/restrictions: The data underlying this article were provided by the Australian Government Department of Human Services and were linked with original data which cannot be shared publicly due privacy protection of the participants. Data will be shared on request to the corresponding author with permission of the Australian Government Department of Human Services. Requests to access these datasets should be directed to https://www.servicesaustralia.gov.au/organisations/about-us/statistical-information-and-data.

## Ethics Statement

The studies involving human participants were reviewed and approved by Barwon Health. The patients/participants provided their written informed consent to participate in this study.

## Author Contributions

BEK, ALS, SEQ, AT, MB, OMD, and LJW conceptualized this study and contributed to the methodology and statistical analyses. SMG contributed to the statistical analyses. JG and KLH-K assisted with data coding. All authors listed have made a substantial, direct and intellectual contribution to the work, and approved it for publication.

## Conflict of Interest

BEK has received travel or grant support from the International Society for the Study of Personality Disorders. JP currently receives funding as a CI for two NHMRC projects (APP1104438, APP1103242), the Norman Beischer Foundation, Amgen and Deakin University. AT has received travel or grant support from the NHMRC, AMP Foundation, National Stroke Foundation, Hunter Medical Research Institute, Helen Macpherson Smith Trust, Schizophrenia Fellowship NSW, SMHR, ISAD, and the University of Newcastle. MB has received Grant/Research Support from the NIH, Cooperative Research Center, Simons Autism Foundation, Cancer Council of Victoria, Stanley Medical Research Foundation, Medical Benefits Fund, National Health and Medical Research Council, Medical Research Futures Fund, Beyond Blue, Rotary Health, A2 milk company, Meat and Livestock Board, Woolworths, Avant and the Harry Windsor Foundation, has been a speaker for Astra Zeneca, Lundbeck, Merck, Pfizer, and served as a consultant to Allergan, Astra Zeneca, Bioadvantex, Bionomics, Collaborative Medicinal Development, Lundbeck Merck, Pfizer and Servier. OMD has received grant support from the Brain and Behavior Foundation, Simons Autism Foundation, Stanley Medical Research Institute, Deakin University, Lilly, NHMRC and Australasian Society for Bipolar and Depressive Disorders (ASBDD)/Servier. HK-H has received grant/research support from University of Eastern Finland and Kuopio University Hospital. LJW has received Grant/Research support from Eli Lilly, Pfizer, The University of Melbourne, Deakin University and the NHMRC. The remaining authors declare that the research was conducted in the absence of any commercial or financial relationships that could be construed as a potential conflict of interest. The handling editor declared a shared affiliation with one of the authors JP.

## References

[1] Volkert J Gablonski T-C Rabung S. Prevalence of personality disorders in the general adult population in Western countries: systematic review and meta-analysis. Br J Psychiatry. (2018) 213:709–15. 10.1192/bjp.2018.202 30261937

[2] Livesley WJ. An empirically-based classification of personality disorder. J Pers Disord. (2011) 25:397–420. 10.1521/pedi.2011.25.3.397 21699399

[3] Gaulin M Simard M Candas B Lesage A Sirois C. Combined impacts of multimorbidity and mental disorders on frequent emergency department visits: a retrospective cohort study in Quebec, Canada. Can Med Assoc J. (2019) 191:E724–32. 10.1503/cmaj.181712 31266786 PMC6606417

[4] Beiser DG Ward CE Vu M Laiteerapong N Gibbons RD. Depression in emergency department patients and association with health care utilization. Acad Emerg Med. (2019) 26:878–88. 10.1111/acem.13726 30884035 PMC6690783

[5] Twomey CD Baldwin DS Hopfe M Cieza A. A systematic review of the predictors of health service utilisation by adults with mental disorders in the UK. BMJ Open. (2015) 5:e007575. 10.1136/bmjopen-2015-007575 26150142 PMC4499684

[6] Quirk SE Berk M Chanen AM Koivumaa-Honkanen H Brennan-Olsen SL Pasco JA. Population prevalence of personality disorder and associations with physical health comorbidities and health care service utilization: a review. Pers Disord Theory Res Treat. (2016) 7:136–46. 10.1037/per0000148 26461047

[7] Rendu A Moran P Patel A Knapp M Mann A. Economic impact of personality disorders in UK primary care attenders. Br J Psychiatry. (2002) 181:62–6. 10.1192/bjp.181.1.62 12091265

[8] Coid J Yang M Bebbington P Moran P Brugha T Jenkins R. Borderline personality disorder: health service use and social functioning among a national household population. Psychol Med. (2009) 39:1721–31. 10.1017/S0033291708004911 19250579

[9] Bender DS Dolan RT Skodol AE Sanislow CA Dyck IR McGlashan TH. Treatment utilization by patients with personality disorders. Am J Psychiatry. (2001) 2:295. 10.1176/appi.ajp.158.2.295 11156814

[10] Britt H Miller GC Henderson J Bayram C Harrison C Valenti L. General Practice Activity in Australia 2015-16. Sydney: Sydney University Press (2016).

[11] Jørgensen JT Andersen JS Tjønneland A Andersen ZJ. Determinants related to gender differences in general practice utilization: Danish Diet, Cancer and Health Cohort. Scand J Primary Health Care. (2016) 34:240–9. 10.1080/02813432.2016.1207141 27421064 PMC5036013

[12] Zimmerman M Rothschild L Chelminski I. The prevalence of DSM-IV personality disorders in psychiatric outpatients. Am J Psychiatry. (2005) 162:1911–8. 10.1176/appi.ajp.162.10.1911 16199838

[13] Pasco JA Nicholson GC Kotowicz MA. Cohort profile: geelong Osteoporosis Study. Int J Epidemiol. (2012) 41:1565–75. 10.1093/ije/dyr148 23283714

[14] Australian Government Department of Health. Medicare Benefits Schedule Book (Operating from 01 January 2014). Canberra, ACT: Australia Government Department of Health (2013).

[15] First MB Spitzer RL Gibbon M Williams JBW. Structured Clinical Interview for DSM-IV-TR Axis I Disorders, Research Version, Patient Edition (SCID-I/P). New York, NY: Biometrics Research; Psychiatric Institute (2002).

[16] First MB Gibbon M Spitzer RL Williams JBW Benjamin LS. Structured Clinical Interview for DSM-IV Axis II Personality Disorders, (SCID-II). Washington, DC: American Psychiatric Association (1997).

[17] Maffei C Fossati A Agostoni I Barraco A Bagnato M Deborah D. Interrater reliability and internal consistency of the structured clinical interview for DSM-IV axis II personality disorders (SCID-II), version 2.0. J Pers Disord. (1997) 11:279–84. 10.1521/pedi.1997.11.3.279 9348491

[18] Zanarini MC Skodol AE Bender D Dolan R Sanislow C Schaefer E. The collaborative longitudinal personality disorders study: reliability of axis I and II diagnoses. J Pers Disord. (2000) 14:291–9. 10.1521/pedi.2000.14.4.291 11213787

[19] Australian Bureau of Statistics. Socio-Economic Indexes for Areas, cat. no. 2033.0.55.001. Canberra, ACT: Australian Bureau of Statistics (2011).

[20] Charlson ME Pompei P Ales KL MacKenzie CR. A new method of classifying prognostic comorbidity in longitudinal studies: development and validation. J Chronic Dis. (1987) 40:373–83. 10.1016/0021-9681(87)90171-8 3558716

[21] StataCorp. Stata Statisical Software: Release 16. College Station, TX: StataCorp. LLC (2019).

[22] Lawn S McMahon J. Experiences of care by Australians with a diagnosis of borderline personality disorder. J Psychiatr Mental Health Nurs. (2015) 22:510–21. 10.1111/jpm.12226 26122817 PMC4755162

[23] Tyrer P Mitchard S Methuen C Ranger M. Treatment rejecting and treatment seeking personality disorders: type R and Type S. J Pers Disord. (2003) 17:263–8. 10.1521/pedi.17.3.263.22152 12839104

[24] Haigh EA Bogucki OE Sigmon ST Blazer DG. Depression among older adults: a 20-year update on five common myths and misconceptions. Am J Geriatr Psychiatry. (2018) 26:107–22. 10.1016/j.jagp.2017.06.011 28735658

[25] Jackson HJ Burgess PM. Personality disorders in the community: a report from the Australian National Survey of Mental Health and Wellbeing. Soc Psychiatry Psychiatr Epidemiol. (2000) 35:531–8. 10.1007/s001270050276 11213842

[26] Penders KA Peeters IG Metsemakers JF Van Alphen SP. Personality disorders in older adults: A review of epidemiology, assessment, and treatment. Curr Psychiatry Rep. (2020) 22:1–14. 10.1007/s11920-020-1133-x 32025914 PMC7002365

[27] Farrer LM Walker J Harrison C Banfield M. Primary care access for mental illness in Australia: Patterns of access to general practice from 2006 to 2016. PLoS ONE. (2018) 13:e0198400. 10.1371/journal.pone.0198400 29856836 PMC5983527

[28] Huang Y-C Lee Y Lin P-Y Hung C-F Lee C-Y Wang L-J. Anxiety comorbidities in patients with major depressive disorder: the role of attachment. Int J Psychiatry Clin Pract. (2019) 23:286–92. 10.1080/13651501.2019.1638941 31464550

[29] Voestermans D Eikelenboom M Rullmann J Wolters-Geerdink M Draijer N Smit JH. The association between childhood trauma and attachment functioning in patients with personality disorders. J Pers Disord. (2020) 12:1–19. 10.1521/pedi_2020_34_474 32163027

[30] Taylor RE Marshall T Mann A Goldberg DP. Insecure attachment and frequent attendance in primary care: a longitudinal cohort study of medically unexplained symptom presentations in ten UK general practices. Psychol Med. (2012) 42:855–64. 10.1017/S0033291711001589 21880165

[31] Byrne M Henagulph S McIvor R Ramsey J Carson J. The impact of a diagnosis of personality disorder on service usage in an adult Community Mental Health Team. Soc Psychiatry Psychiatr Epidemiol. (2014) 49:307–16. 10.1007/s00127-013-0746-3 23959588

[32] Fok ML-Y Stewart R Hayes RD Moran P. Predictors of natural and unnatural mortality among patients with personality disorder: evidence from a large UK case register. PLoS ONE. (2014) 9:e0100979. 10.1371/journal.pone.0100979 25000503 PMC4085063

